# Analysis of Kallikrein 6, Acetyl-α-Tubulin, and Aquaporin 1 and 2 Expression Patterns During Normal Human Nephrogenesis and in Congenital Anomalies of the Kidney and Urinary Tract (CAKUT)

**DOI:** 10.3390/genes16050499

**Published:** 2025-04-27

**Authors:** Nela Kelam, Marin Ogorevc, Ivona Gotovac, Ivana Kuzmić Prusac, Katarina Vukojević, Mirna Saraga-Babić, Snježana Mardešić

**Affiliations:** 1 Laboratory for Early Human Development, Department of Anatomy, Histology and Embryology, University of Split School of Medicine, Šoltanska 2A, 21000 Split, Croatia; nela.kelam@mefst.hr (N.K.); katarina.vukojevic@mefst.hr (K.V.); smardesi@mefst.hr (S.M.); 2 Department of Pathology, University Hospital Split, Spinciceva 1, 21000 Split, Croatia; mogorevc@mefst.hr (M.O.); ivanakp@mefst.hr (I.K.P.); 3 Clinical Department of Diagnostic and Interventional Radiology, University Hospital Centre Split, Spinciceva 1, 21000 Split, Croatia; ivona.kosovic@mefst.hr; 4 Center for Translational Research in Biomedicine, University of Split School of Medicine, Šoltanska 2A, 21000 Split, Croatia; 5 Mediterranean Institute for Life Sciences, University of Split, Meštrovićevo Šetalište 45, 21000 Split, Croatia

**Keywords:** kallikrein-related peptidase 6, human, acetyl-α-tubulin, AQP1, AQP2, nephrogenesis, congenital anomalies of kidney and urinary tract, CAKUT

## Abstract

**Background/Objectives**: The human kallikrein-related peptidase 6 (KLK6), a serine protease with trypsin-like properties, belongs to the 15-member kallikrein (*KLK*) gene family and is predominantly recognized for its role in oncogenesis, neurodegenerative disorders, and skin conditions. Aquaporins (AQPs) are integral membrane proteins that facilitate water transport across cell membranes. AQP1 is constitutively active in the kidneys and plays a crucial role in reabsorbing filtered water, while AQP2 is regulated by vasopressin and is essential for maintaining body fluid homeostasis. The primary objective of the present study is to investigate the spatio-temporal expression patterns of KLK6, AQP1, and AQP2 throughout normal human nephrogenesis and congenital kidney and urinary tract (CAKUT) abnormalities: duplex kidneys, horseshoe kidneys, and dysplastic kidneys. **Methods**: An immunofluorescence analysis of KLK6, AQP1, and AQP2 was performed on 37 paraffin-embedded fetal kidney samples. The area percentage of KLK6 in the kidney cortex was calculated in normal developing samples during developmental phases 2, 3, and 4 and compared with CAKUT samples. **Results**: KLK6 exhibits distinct spatiotemporal expression patterns during human kidney development, with consistent localization in proximal tubules. Its subcellular positioning shifts from the basolateral cytoplasm in early phases to the apical cytoplasm in later stages, which may be strategically positioned to act on its substrate in either the peritubular space or the tubular fluid. KLK6 expression followed a quadratic trajectory, peaking at Ph4. This marked increase in the final developmental phase aligns with its strong expression in mature kidneys, suggesting a potential role in proximal tubule differentiation and functional maturation through facilitating extracellular matrix remodeling and activating proteinase-activated receptors, modulating the signaling pathways that are essential for tubular development. In duplex kidneys, structural abnormalities such as ureteral obstruction and hydronephrosis may upregulate KLK6 as part of a reparative response, while its downregulation could impair epithelial remodeling and cytoskeletal integrity, exacerbating dysplastic phenotypes. **Conclusions**: These findings highlight the potential of KLK6 involvement in normal kidney development and the pathology of CAKUT.

## 1. Introduction

Kidney development is a highly coordinated process that depends upon precise molecular crosstalk between distinct cellular compartments to ensure proper organ formation. The intricate signaling interactions between the ureteric bud and the metanephric mesenchyme during these critical early stages are indispensable in appropriate kidney morphogenesis and functional maturation [1,2,3]. These interactions are mediated by a variety of molecular pathways, many of which converge on the primary cilium, a sensory microtubule-based organelle on the surface of epithelial cells that plays a pivotal role in regulating key developmental processes, such as cell proliferation, differentiation, and tissue patterning [4,5,6]. Disruptions in ciliary function, often caused by mutations in genes encoding ciliary proteins, give rise to a group of disorders collectively termed ciliopathies [7]. Ciliopathies have been increasingly implicated in the pathogenesis of congenital anomalies of the kidney and urinary tract (CAKUT) and some other systemic conditions [4,7], as the primary cilium is essential for mediating signal transduction pathways, including Hedgehog, Wnt, and Notch signaling, which are critical for normal nephrogenesis [6,8]. Consequently, defects in ciliary function can lead to impaired ureteric bud branching, aberrant nephron induction, and dysregulated renal differentiation, culminating in a spectrum of structural and functional abnormalities that are characteristic of CAKUT [9].

CAKUT represents a leading cause of chronic kidney disease (CKD) in the pediatric population, accounting for approximately 40–50% of cases [10]. According to the 2024 EUROCAT guidelines, CAKUT is categorized into eight subgroups, including three newly introduced classifications: (a) unilateral renal agenesis, (b) bilateral renal agenesis/Potter sequence, (c) multicystic renal dysplasia, (d) congenital hydronephrosis/ureteral obstruction, (e) ectopic or lobulated kidneys, (f) bladder exstrophy/epispadias, (g) posterior urethral valves, and (h) prune belly syndrome [11], with disease severity varying from asymptomatic conditions to life-threatening complications [10]. Severe phenotypes often result from disrupted signaling during early nephrogenesis, potentially leading to obstructive uropathies, profound renal dysfunction, or end-stage kidney failure [12]. Significantly, CAKUT is associated with an elevated risk of mortality, as affected infants are more likely to experience severe morbidity or death compared with those without such anomalies [13].

The human kallikrein-related peptidase 6 (KLK6), a serine protease with trypsin-like properties, belongs to the 15-member kallikrein (*KLK*) gene family mapping to chromosome 19q13.3–13.4 [14]. *KLK6* is highly expressed in the brain, kidney, and pancreas [15,16,17]. This gene is predominantly recognized for its role in oncogenesis [15,18,19,20], neurodegenerative disorders [21,22,23], and skin conditions [22]. Emerging evidence suggests potential associations with renal pathophysiology, like renal cell carcinoma (RCC) [24,25], though its functional significance in the kidney remains less characterized compared with other kallikreins, such as tissue kallikrein (KLK1) [26]. Gabril et al. reported strong cytoplasmic KLK6 expression in the proximal and distal tubules, weak expression in the mesangium, and no expression in Bowman’s capsule in the normal human kidney [24]. While the classical kallikrein–kinin system (KKS), primarily involving KLK1, is well established in renal homeostasis—regulating blood pressure, sodium–potassium balance, and cytoprotective pathways via nitric oxide and prostaglandins—the direct involvement of KLK6 in these processes is less defined [27,28]. In contrast, KLK1 is synthesized in renal tubular cells and modulates ion transport in the distal tubules, underscoring the functional diversity within the kallikrein family [29,30].

α-Tubulin is a fundamental component of the eukaryotic cytoskeleton, forming microtubules by assembling with β-tubulin into heterodimers. The gene encoding α-tubulin (*TUBA1A*) is conserved across all eukaryotes and plays a critical role in microtubule polymerization, ensuring proper cell division, signaling, and structural integrity [31]. The acetylation of α-tubulin, a conserved post-translational modification of the fundamental component of primary cilia, plays a pivotal role in microtubule stability and ciliary function, which are essential for renal nephrogenesis. This modification is associated with the formation of stable microtubules that support intracellular transport, structural organization, and, consequently, the proper function of primary cilia [32,33]. Our previous studies have demonstrated that α-tubulin is strongly expressed throughout kidney development, marking the presence of primary cilia in all developing structures. Notably, it is prominently localized on the surfaces of the parietal epithelial cells of Bowman’s capsule, as well as in the glomerulus, proximal convoluted tubules, and distal convoluted tubules of the human kidney [34]. Another study has confirmed that the dysregulation of tubulin acetylation is implicated in CAKUT, where defective ciliary signaling disrupts epithelial differentiation and tissue morphogenesis [34,35], as well as in ciliopathies like polycystic kidney disease (PKD) [36,37]. A study by Manissorn et al. has demonstrated that α-tubulin acetylation enhances tissue repair and reduces cytotoxicity in renal tubular cells exposed to stressors, highlighting its protective role in the kidney [38].

Aquaporins (*AQP*), found in all living organisms from bacteria to humans, are key water channel proteins that play vital roles in renal water transport driven by osmotic forces and homeostasis. A total of 13 isoforms have been identified in human tissue, with at least 8 found in the kidney. In humans, AQP1 is highly expressed in the proximal tubules and descending thin limbs of Henle’s loop, facilitating efficient water reabsorption and contributing to the countercurrent mechanism that is essential in urine concentration [39,40]. AQP2, which is mostly expressed in the principal cells of the collecting duct and connecting tubule in mature kidneys, is regulated by vasopressin and mediates water reabsorption through dynamic trafficking between intracellular vesicles and the apical plasma membrane [39,40,41,42]. Both aquaporins are present early in nephrogenesis, indicating their significance in kidney development. AQP1 is first observed in developing human kidneys proximal tubules at 12 weeks of gestation and later appears in the descending thin limbs by 15 weeks; meanwhile, AQP2 is detected as early as 12 weeks in the ureteric bud and collecting duct system, emphasizing their essential roles in kidney formation [39,40]. It has been demonstrated that AQP2-positive cells in the embryonic kidney act as progenitor cells for the collecting duct, connecting tubule, and certain distal convoluted tubule cells [43]. The dysregulation of AQP1 or AQP2 has been implicated in CAKUT and other renal disorders, such as autosomal dominant PKD (ADPKD) [39]. In ADPKD, altered localization or reduced expression of AQP1 and AQP2 disrupts standard water transport mechanisms, contributing to cyst formation and progression. Furthermore, studies have highlighted the role of AQP2 in water-balance disorders, including nephrogenic diabetes insipidus and post-obstructive polyuria [44,45].

The primary objective of the present study is to investigate the spatial and temporal expression pattern of KLK6, as well as AQP1 and AQP2, in the cortex of the kidney throughout normal human nephrogenesis and CAKUT: duplex kidneys, horseshoe kidneys, and dysplastic kidneys. Our earlier studies examined the different CAKUT candidate genes and signaling pathways implicated in nephrogenesis [46,47]. Despite extensive research into the various genetic factors contributing to CAKUT, there is a notable lack of studies focusing on KLK6 expression in this context. Given the involvement of the observed protein in tissue remodeling and its potential role in regulating key biological processes, understanding its expression in renal development and different CAKUT phenotypes could provide novel insights into the molecular mechanisms driving kidney formation and dysfunction. By addressing this gap in the literature, this study aims to evaluate KLK6 as a potential biomarker for early diagnosis or as a novel therapeutic target, which could lead to improved clinical management and outcomes in patients with congenital kidney disorders.

## 2. Materials and Methods

### 2.1. Procurement and Processing of Tissues

In total, 37 paraffin-embedded tissue blocks containing fetal kidney samples (Table 1) were collected from the University Hospital of Split, Department of Pathology. These specimens were obtained from cases of spontaneous miscarriage and eugenic abortions associated with severe congenital abnormalities. The study included only well-preserved, non-macerated samples to ensure the integrity of the histopathological analysis.

This same tissue cohort was previously utilized in our earlier studies examining the expression patterns of selected CAKUT-related genes during human kidney development [46,47]. Although these studies share a common tissue cohort, the experimental design, analytical focus, and biological targets differ significantly.

The collection and processing of the tissue samples were conducted with ethical approval from the Ethics and Drug Committee of the University Hospital of Split (class: 003-08/23-03/0015, protocol code no.: 2181-198-03-04-23-0073, date of approval: September 27, 2023), following the ethical principles outlined in the Declaration of Helsinki [48]. The gestational age was estimated using both maternal menstrual history and external fetal measurements, specifically crown–rump length [49], to provide accurate developmental staging.

Renal pathology classification was performed through routine histopathological examination and gross morphological assessment to ensure a detailed evaluation of the kidney development and structural anomalies across the fetal stages examined.

Fetal kidney tissue was fixed in 4% paraformaldehyde prepared in 0.1 M phosphate-buffered saline (PBS) after post-mortem dissection and subsequently embedded in paraffin blocks, as previously outlined [46,47]. The tissue was cut into serial sections, with every 10th section stained using hematoxylin–eosin to verify adequate tissue preservation. Light microscopy was employed to assess the stages of normal fetal kidney development and to identify pathological changes in the kidneys affected by CAKUT.

### 2.2. Immunofluorescence Staining

Immunofluorescence staining was carried out following the protocol previously described [35,50,51]. Briefly, after deparaffinization and rehydration, antigen retrieval was conducted in a 0.01 M citrate buffer using a heat steamer for 30 min. The samples were then incubated overnight at room temperature with primary antibodies in a humidity chamber (Table 2). The following day, after PBS rinsing, secondary antibodies were applied for two hours at room temperature, followed by additional PBS washes (Table 2). DAPI (4′,6-diamidino-2-phenylindole) staining was used as a nuclear counterstain to facilitate tissue orientation and morphological assessment during imaging, as reported previously [46,47]. It was not used for the quantitative analysis and did not directly contribute to interpreting KLK6 expression. Slides were mounted with Immuno-Mount (Thermo Shandon, Pittsburgh, PA, USA) prior to coverslipping.

Isotype-matched controls and secondary-only samples were utilized to diminish nonspecific background signals. Isotype-matched controls entailed replacing the primary antibody with a non-target-specific antibody of the same isotype, facilitating the evaluation of probable nonspecific binding. In the secondary-only controls, the primary antibody was excluded to detect any nonspecific secondary antibody binding. These controls ensured that the detected signals were specific to the target protein and were not influenced by background noise.

### 2.3. Data Acquisition

Fluorescence imaging was conducted using an Olympus BX51 epifluorescence microscope (Tokyo, Japan) equipped with a Nikon DS-Ri2 camera (Nikon Corporation, Tokyo, Japan) and operated using the NIS-Elements F software (version 5.22.00). Images were acquired at 40× magnification with standardized gain and exposure settings. The expression of KLK6 was analyzed in a minimum of ten representative fields of view from the nephrogenic zone and the juxtamedullary area of the cortex of normal developing (CTRL), horseshoe (HK), duplex (DK), and dysplastic (DYS) kidneys. Positive KLK6 staining appeared as a diffuse, granular, or punctate green signal.

### 2.4. Image Analysis of the Area Percentage

The acquired microphotographs were examined using the ImageJ software version 1.54g (National Institutes of Health, Bethesda, MD, USA) to quantify the immunofluorescence signals of the detected proteins [46,51,52]. Each image underwent the subsequent processing procedure: fluorescence leakage was reduced by eliminating the red counter-signal from the green fluorescence channel. A median filter with a radius of 9.0 pixels was subsequently applied. The unfiltered photos were deducted from the filtered images to isolate the positive signals, and the resultant images were transformed into an 8-bit format. Subsequently, each image was systematically altered by employing the triangle thresholding algorithm. The fluorescence area percentage was ascertained utilizing the “analyze particles” function.

Three experienced histologists analyzed the microphotographs independently and established the background thresholds using negative controls to account for observer variability. Interrater agreement was assessed via an intraclass correlation analysis, yielding a coefficient of >0.8, indicating a strong concordance [53].

### 2.5. Statistical Analysis of the Area Percentage

The statistical analysis was performed using the GraphPad Prism version 9.0.0 software (GraphPad Software, San Diego, CA, USA). The data were first averaged for each examined phenotype.

The results are presented as the mean and standard deviation of the calculated percentages. The normality of the data distribution was evaluated using the Shapiro–Wilk test. Every dataset related to the area percentage analysis had a probability level (*p*) of less than 0.05, which was considered statistically significant.

KLK6 expression levels within the cortical region of healthy control kidneys across developmental phases 2, 3, and 4 were evaluated and compared using a standard one-way analysis of variance (ANOVA), followed by Tukey’s multiple comparisons test.Linear and nonlinear regression modeling was employed to examine the dynamics and trends of KLK6 expression throughout the developmental stages of healthy control kidneys. We computed the average area % values of the kidney cortex in samples of various developmental stages. A coefficient is defined as a point estimate plus or minus the standard error in the trend description models. The coefficient of determination (R^2^) functioned to evaluate the goodness of fit.

We examined the area percentages of the cortex of the fetal kidneys, contrasting healthy kidneys with those affected by CAKUT (DK, HK, and DYS) using a one-way ANOVA followed by a Tukey’s post-hoc analysis.

The graphs have been designed utilizing GraphPad Prism, while the figure panels have been assembled using Adobe Photoshop (version 21.0.2). The microphotographs underwent background removal and contrast augmentation to enhance the visibility and clarity.

## 3. Results

### 3.1. Normal Human Kidney Development

The development of the human definitive kidney (metanephros) begins in the 5th developmental week when the subsequent division of the ureteric bud induces nephron formation in the nearby metanephrogenic blastema. Kidney growth is executed through the 15 successive generations of nephrons at the periphery of the developing kidney, with the outermost nephrons less developed than those positioned more centrally. The overall development of the metanephros can be divided into four stages [54,55]. The first stage lasts from the 5th to the 14th developmental week and is characterized by the most intense division of the metanephric duct and nephron induction. In the second stage (the period between the 15th and 22nd developmental week), the cessation of the nephron induction is observed. A further decrease in nephron formation characterizes the third period (22nd to 32nd/36th developmental week). In contrast, in the fourth period, which lasts from the 36th developmental week into childhood, only interstitial growth and cell differentiation are observed in developing nephrons.

#### 3.1.1. Double Immunofluorescence Staining with Kallikrein 6 (KLK6) and Acetyl-α-Tubulin During Normal Human Kidney Development

In the second period (15th and 17th developmental weeks), the kidney cortex contains well-developed nephrons consisting of glomeruli, proximal, and distal convoluted tubules, and interstitial connective tissue with blood vessels. Parts of Henley’s loop and the collecting system are found in the centrally positioned kidney medulla.

**Figure 1:**
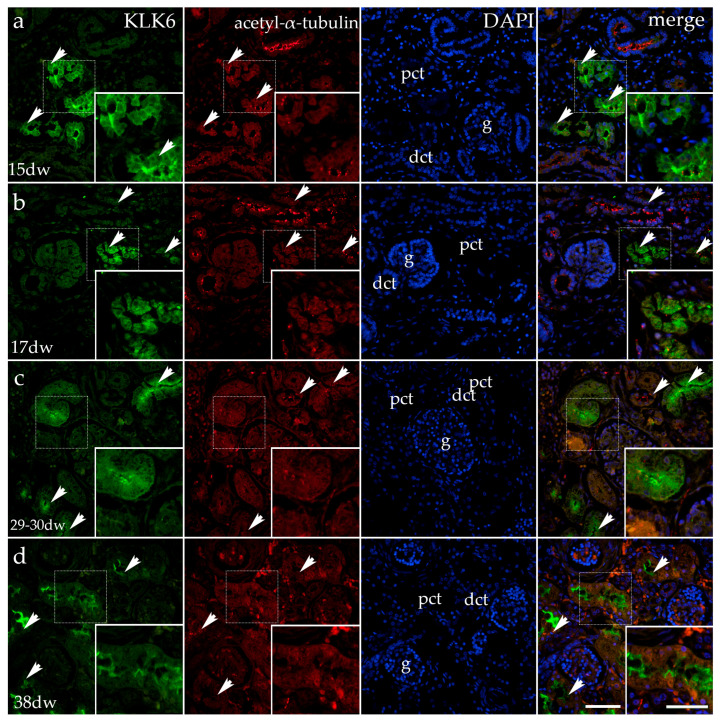
Immunofluorescence staining of the renal cortex of a normal human fetal kidney (CTRL) at developmental phase 2 (15th and 17th developmental week (dw); (**a**,**b**)), developmental phase 3 (29–30th dw; (**c**)) and developmental phase 4 (38th dw; (**d**)), with the antibody for kallikrein 6 (KLK6; green) and acetyl-α-tubulin (red). The arrows indicate the expression pattern of KLK6 and acetyl-α-tubulin in the glomeruli (g), proximal convoluted tubules (pcts), and distal convoluted tubules (dcts) of the renal cortex, indicated on the 4′,6-diamidino-2-phenylindole staining image (DAPI). DAPI staining (blue) was used to visualize the nuclei and aid in tissue orientation. It serves solely as a structural reference and is not involved in the analysis of KLK6 expression. The immunoexpression of merged KLK6, acetyl-α-tubulin, and DAPI in a normal human fetal kidney (merge; orange). The inserts that match the dashed boxes indicate the major region of protein expression. The scale bar is 50 μm for all the images.

KLK6-positive cells characterize proximal kidney tubules made of cuboidal cells with brush borders, while the remaining kidney structures are negative for KLK6. In the 15th week, the signal is moderate in the basal and lateral cell cytoplasm of the proximal tubules, while it is strong at the apical cell surface. In the 17th week, the intensity of the signal increases in the proximal tubules and has a granular appearance. When stained with acetyl-α-tubulin (characteristic in primary cilia), the elongated cilia become visible on the apical surfaces of all the tubular cells and glomeruli, which are particularly numerous in the distal tubules. DAPI staining visualizes the cell nuclei with a blue stain. The merging of the images reveals the co-expression of KLK6 and acetyl-α-tubulin at the apical surfaces of proximal tubular cells (Figure 1a,b).

In the third period of kidney development (29th–30th week), the cell cytoplasm of the proximal tubular cells becomes cylindrical, while the lumen is narrow and irregular. KLK staining is mild in the cell cytoplasm of proximal tubules, except for its apical part, which shows a strong granular signal.

A somewhat less intense signal characterizes the primary cilia when stained with acetyl-α-tubulin. When merging all the images (including the nuclear DAPI stain), the co-expression of KLK6 and acetyl-α-tubulin is observed in the proximal tubular cells (Figure 1c).

In the fourth developmental period of kidney development (38th week), the cells of the proximal tubules are very high, and they enclose an irregular lumen, which is primarily caused by the dense apical microvilli that form the brush border. KLK6 staining is seen only in the proximal tubules, but now the signal is reduced to the most apical part of the cell, while it is missing in the remaining basal and lateral cytoplasm. Like in the previous period, the acetyl-α-tubulin signal is less intense than in the previous developmental stages. When merging the images, the co-expression of KLK6 and acetyl-α-tubulin is observed in the proximal tubular cells (Figure 1d).

#### 3.1.2. Double Immunofluorescence Staining of KLK6, Aquaporin 1 (AQP1), and Aquaporin 2 (AQP2) in Developing Normal Human Kidneys

Throughout human kidney development, there is a characteristic change in KLK6 expression (as already described for the KLK6 and acetyl-α-tubulin co-expression in Figure 1).

**Figure 2:**
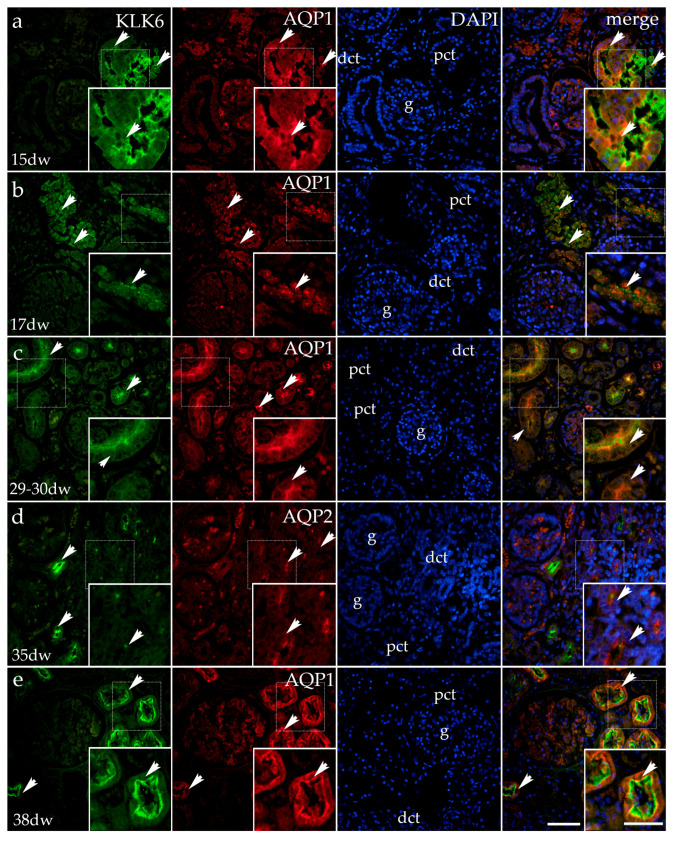
Immunofluorescence staining of the renal cortex of a normal human fetal kidney (CTRL) at developmental phase 2 (15th and 17th developmental week (dw); (**a**,**b**), developmental phase 3 (29–30th and 35th dw; (**c**,**d**) and developmental phase 4 (38th dw); (**e**)), with the antibody for kallikrein 6 (KLK6; green) aquaporin 1 and aquaporin 2 (AQP1 and AQP2 red). The arrows indicate the pattern of expression of KLK6 and AQP1 or AQP2 in the glomeruli (g), proximal convoluted tubules (pcts), and distal convoluted tubules (dcts) of the renal cortex, indicated on the 4′,6-diamidino-2-phenylindole staining image (DAPI). DAPI staining (blue) was used to visualize the nuclei and aid in tissue orientation. The immunoexpression of merged KLK6, AQP1 or AQP2, and DAPI in a normal human fetal kidney (merge; orange). The inserts that match the dashed boxes indicate the major region of protein expression. The scale bar is 50 μm for all the images.

In the 15th developmental week, AQP1 staining characterizes the basal and apical parts of the proximal tubules and the walls of blood vessels. The merging of the images representing KLK6, AQP1, and the DAPI nuclear stain reveals partial co-expression of KLK6/AQP1 in the apical cytoplasm of the proximal tubules. Although expressed in the same cells of proximal tubules, KLK6 and AQP1 are localized in different cytoplasmic compartments (Figure 2a).

In the 17th developmental week, AQP1 expression is observed in the form of coarse grains in the cytoplasm of proximal tubules and the glomeruli (the blood vessel wall). Following the merging of the images (KLK6, AQP1, and DAPI), the partial co-expression of KLK6/AQP1 is observed only in some cytoplasmic parts of the proximal tubules. The remaining parts of the cytoplasm are occupied either by KLK6 or AQP1 grains, while the glomeruli show only AQP1 expression (Figure 2b).

In the 29th–30th developmental week, in comparison with the earlier developmental stages, AQP1 expression changes and is now found at the apical surfaces of the proximal tubule cells and as fine grains in the wall of the glomerular blood capillaries. The merging of the KLK6, AQP1, and DAPI images shows only small cytoplasmic areas of overlapping expression, as the two antibodies localize in different cytoplasmic compartments (Figure 2c).

In the 35th developmental week, the AQP2 signal in the developing human kidneys is localized in the apical cytoplasm of some distal tubules in the form of coarse granules. After merging the KLK6/AQP2 antibodies and the DAPI nuclear stain, the co-localization of KLK6 and AQP2 is mostly not observed, although some cells in the distal tubules show a very weak KLK6 signal in parallel with the AQP2 signal (Figure 2d).

In the 38th developmental week, the expression of KLK6 is observed in the apical third of the proximal tubular cells, while AQP1 characterizes the lower basal two-thirds of their cytoplasm. After merging the images with DAPI, the co-expression of KLK6 and AQP1 is observed as a thin borderline expression. The remaining tubular cell cytoplasm is occupied by KLK6 in the apical compartment and by AQP1 in its basal compartments. AQP1 is also observed in the glomerular capillaries (Figure 2e).

#### 3.1.3. Double Fluorescence Staining of KLK6, Acetyl-α-Tubulin, AQP1, and AQP2 in Postnatal Normal Human Kidneys

In healthy postnatal kidneys (1.5 years old), KLK6 is strongly expressed in the apical cytoplasm of the proximal tubules, while its expression is absent in all other parts of the kidney tissue.

**Figure 3:**
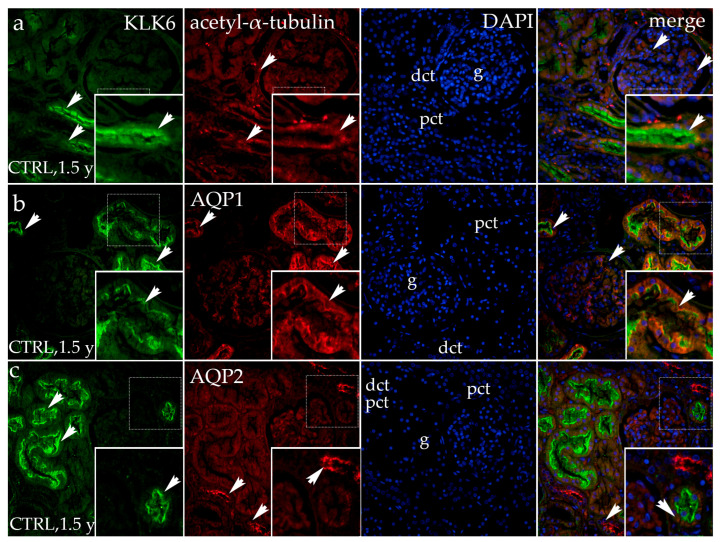
Immunofluorescence staining of the renal cortex of a normal postnatal human kidney (CTRL) at developmental phase 4 (1.5 year (y)) with the antibody for kallikrein 6 (KLK6; green), acetyl-α-tubulin (**a**), aquaporin 1 (AQP1; red, (**b**), and aquaporin 2 (AQP2; red, (**c**). The arrows indicate the pattern of expression of KLK6, acetyl-α-tubulin, AQP1, and AQP2 in the glomeruli (g), proximal convoluted tubules (pcts), and distal convoluted tubules (dcts) of the renal cortex indicated on the 4′,6-diamidino-2-phenylindole staining image (DAPI). DAPI staining (blue) was used to visualize the nuclei and aid tissue orientation. The immunoexpression of merged KLK6, acetyl-α-tubulin, AQP1, AQP2, and DAPI in a normal human fetal kidney (merge; orange). The inserts that match the dashed boxes indicate the major region of protein expression. The scale bar is 50 μm for all the images.

The expression of acetyl-α-tubulin is observed in the cytoplasm of the proximal tubules (corresponding to the basal bodies/centrioles) and in the primary cilia of the apical surface of the proximal and distal convoluted tubules and glomeruli. The merging of the images of KLK6, acetyl-α-tubulin, and the DAPI nuclear stain shows the co-expression of KLK6 and acetyl-α-tubulin in places where the primary cilia extend into the lumen (Figure 3a).

In the same healthy postnatal kidney tissue, KLK6 staining shows the same pattern, while AQP1 expression is observed throughout the cytoplasm of the proximal tubules, showing different intensities of the signal, but being particularly strong at its apical surface. The merging of the two signals with DAPI reveals only partial co-expression of the two antibodies, which intermingles with the areas of non-existing co-expression, in which each antibody has its own compartment of apical cell cytoplasm (Figure 3b).

The double immunofluorescence staining of healthy postnatal kidneys with KLK6 and aquaporin 2 (AQP2) shows an intense AQP2 signal in the form of coarse granules localized at the apical surface of distal tubules and/or connecting tubules. The merging of the images shows no co-expression of KLK6 and the AQP2 signal (Figure 3c).

### 3.2. Double Fluorescence Staining of KLK6 with Acetyl-α-Tubulin, AQP1, and AQP2 Antibodies in the Horseshoe and Duplex Human Kidneys

Horseshoe kidneys are characterized as having functioning renal masses that are present on both sides of the vertebral column, connected by an isthmus of renal tissue or by fibrous tissue.

**Figure 4:**
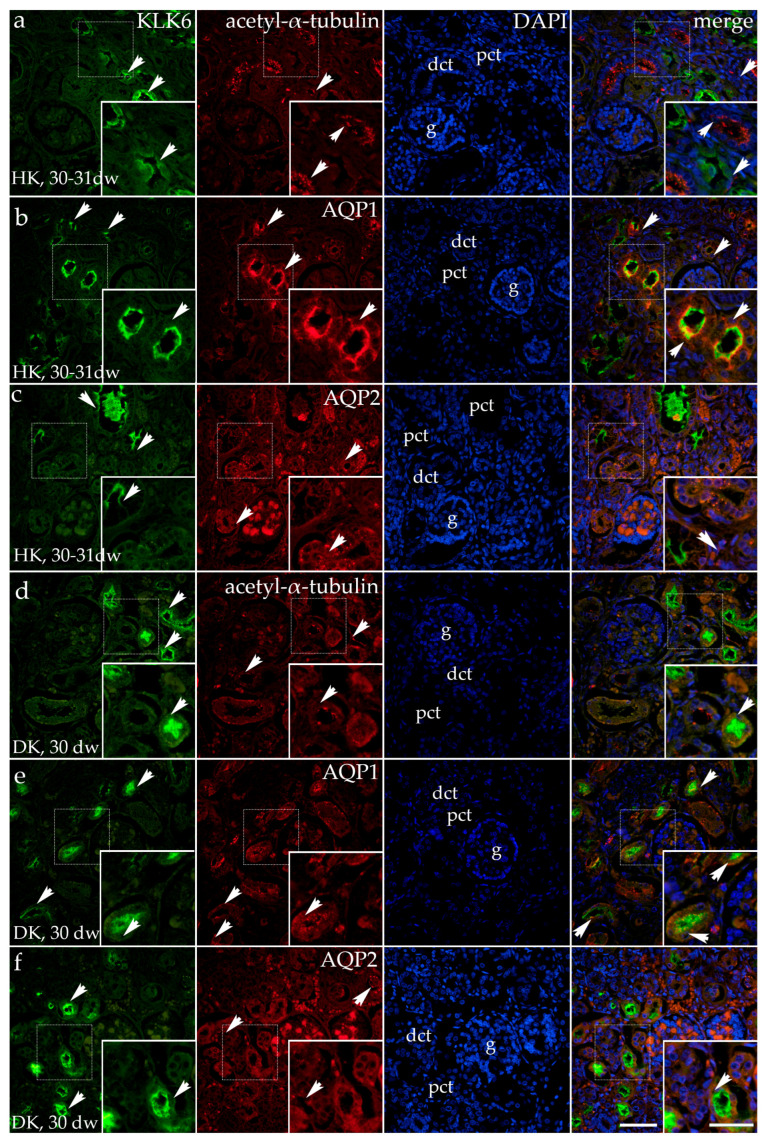
Immunofluorescence staining of the renal cortex of the horseshoe kidneys (HKs) at the 30th–31st developmental week (dw) (**a**–**c**) and duplex kidneys (DKs) at the 30th dw (**d**–**f**) with the antibody for kallikrein 6 (KLK6; green), acetyl-α-tubulin, and aquaporin 1 (AQP1) or aquaporin 2 (AQP2; red). The arrows indicate the pattern of expression of KLK6, acetyl-α-tubulinAQP1, and AQP2 in the glomeruli (g), proximal convoluted tubules (pcts), and distal convoluted tubules (dcts) of the renal cortex, indicated on the 4′,6-diamidino-2-phenylindole staining image (DAPI). DAPI staining (blue) was used to visualize the nuclei and aid in tissue orientation. The immunoexpression of merged KLK6, acetyl-α-tubulin, AQP1, AQP2 and DAPI in a normal human fetal kidney (merge; orange). The inserts that match the dashed boxes indicate the major region of protein expression. The scale bar is 50 μm for all the images.

In the tissue of the horseshoe kidneys in the 30th to 31st developmental week (third developmental period), the cortex contains the proximal tubules with a dilated and irregular lumen, the glomeruli appear lobulated, while the distal tubules show no significant morphological changes. The KLK6 signal observed in the cytoplasm of the proximal tubules appears irregular, with the strongest expression at its apical surface. Acetyl-α-tubulin staining in the primary cilia was observed in all parts of the nephron, rarely in the proximal tubules but particularly long, branching, and irregular in the distal tubules. The merging of KLK6 and acetyl-α-tubulin with DAPI shows rare co-expression of KLK6 and acetyl-α-tubulin in the proximal tubules because of the absence of the primary cilia in the dilated proximal tubule (Figure 4a).

Besides KLK6, AQP1 also shows an irregular distribution in the dilated proximal tubules of horseshoe kidneys. The AQP1 signal is observed at the basal, lateral, and apical surfaces of the proximal tubular cells and is present in the form of grains within the glomeruli and blood vessels. The merging of the images shows co-expression of KLK6 and AQP1 in the apical cytoplasm of the proximal tubules (Figure 4b).

KLK6/AQP2 staining of the same horseshoe kidneys shows an irregular apical expression of KLK6 in some cysts of the proximal tubules, with a strong signal observed in the lumen resulting from the shedding of the apical cytoplasm in the proximal tubular cells. The granular expression of AQP2 is observed in the basal and, in some cases, in the apical cytoplasm of the dilated proximal tubules, and as coarse granules in the cytoplasm of the distal tubules, as well as a weak signal in the glomeruli. The merging of KLK6, AQP2, and the DAPI nuclear stain shows the absence of the co-expression of KLK6 and AQP2 (Figure 4c).

In the tissue of the duplicated kidney (30 dw, third developmental period), we found expression of KLK6 and acetyl-α-tubulin to have a nearly typical expression pattern: KLK6 expression localized in the apical cytoplasm of proximal kidney tubules, while acetyl-α-tubulin-stained primary cilia in all kidney structures. Merging of the two antibodies with DAPI nuclear stain reveals co-expression of KLK6 and acetyl-α-tubulin in the proximal tubule, while the strong KLK6 signal often covered the thin signal of primary cilia or basal bodies (Figure 4d).

Staining of the duplicated kidney (30 developmental weeks) with KLK6 and AQP1 shows a typical expression of KLK6 in the apical cytoplasm of the proximal tubules, while AQP1 shows basal granular expression in some proximal tubules, and other proximal tubules display both apical and basal granular expression. The merging of the images reveals the co-expression of KLK6 and AQP1 in the apical cytoplasm of some proximal tubules and its absence in tubules where KLK6 expression is missing, while AQP1 expression is localized basally (Figure 4e).

In the same tissue of the duplicated kidneys, some proximal tubules have a regular appearance and expression of KLK6 in the apical cytoplasm, while the other slightly dilated proximal tubules show irregular expression. A mild, granular AQP2 signal is observed primarily in the distal tubules and glomeruli, and its signal is less intense than in normal developing kidneys. The merging of KLK6 and AQP2 shows an absence of their co-expression (Figure 4f).

### 3.3. Dysplastic Kidneys

Dysplastic kidneys are described as a variety of disorders associated with abnormally developed kidney tissue, usually connected to poorly differentiated nephrons and collecting ducts, as well as increased stroma. Cysts and metaplastic tissues can also be occasionally found.

#### Double Fluorescence Staining of KLK6, Acetyl-α-Tubulin, and AQP1 in the Dysplastic and Hypoplastic Human Fetal Kidneys

**Figure 5:**
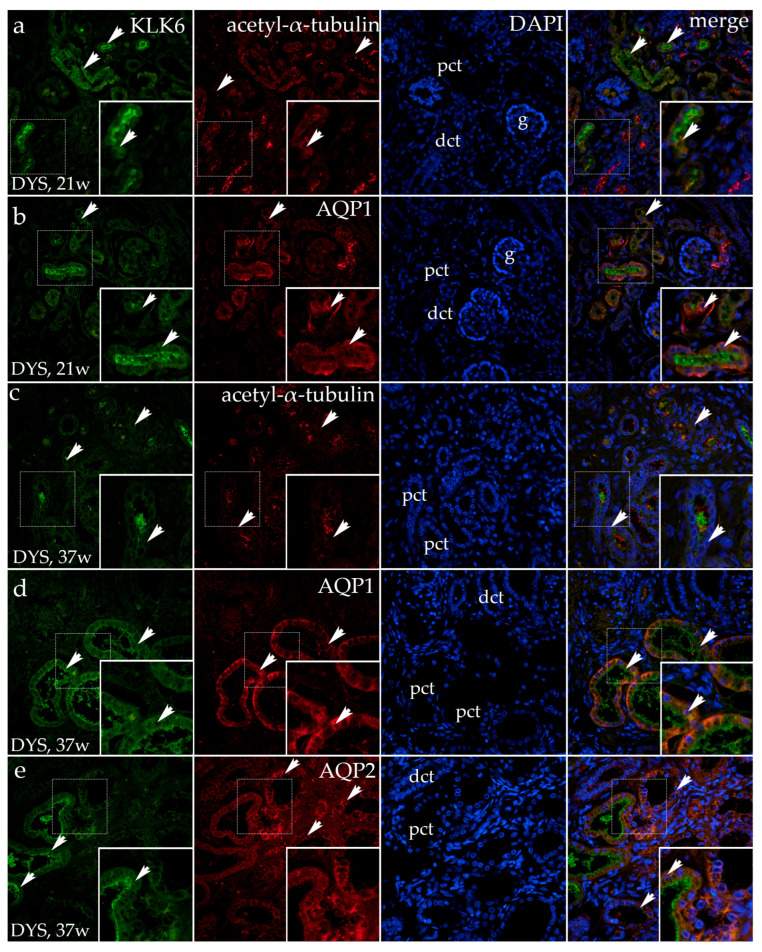
Immunofluorescence staining of the renal cortex of the dysplastic kidneys (DYSs) at the 21st developmental week (dw, (**a**,**b**)) and the 37th dw (**c**–**e**) with the antibody for kallikrein 6 (KLK6; green), acetyl-α-tubulin, and aquaporin 1 (AQP1) or aquaporin 2 (AQP2; red). The arrows indicate the pattern of expression of KLK6, acetyl-α-tubulin, AQP1, and AQP2 in the glomeruli (g), proximal convoluted tubules (pcts), and distal convoluted tubules (dcts) of the renal cortex, indicated on the 4′,6-diamidino-2-phenylindole staining image (DAPI). DAPI staining (blue) was used to visualize the nuclei and aid in tissue orientation. The immunoexpression of merged KLK6, acetyl-α-tubulin, AQP1, AQP2, and DAPI in a normal human fetal kidney (merge; orange). The inserts that match the dashed boxes indicate the major region of protein expression. The scale bar is 50 μm for all the images.

In the 21st developmental week, the human kidneys are at the end of the second developmental period, containing proximal tubules that show strong KLK6 positivity at their apical part, while the distal tubules appear negative for the KLK6 antibody. Acetyl-α-tubulin stains the primary cilia along the different nephron parts. The primary cilia are extremely developed in the distal tubules. The merging of the images of KLK6, acetyl-α-tubulin, and the DAPI nuclear stain shows the co-expression of KLK6 and primary cilia at the apical cell surfaces of the proximal tubular cells (Figure 5a).

When the same dysplastic kidneys are stained with KLK6 and AQP1, moderate-to-strong expression of AQP1 characterizes only the basal parts of the proximal tubular cells, but it is strong in the walls of blood vessels. The merging of different images (KLK6, AQP1, and DAPI) shows the presence of both KLK6 and AQP1 in the proximal tubular cells. However, they localize in different cytoplasmic compartments. In contrast to the normal kidney tissue in the second period of kidney development, the KLK6 signal appears only in the apical cytoplasm of the proximal tubules. In contrast to normal kidneys, in dysplastic kidneys, the AQP1 signal is expressed only in the basal cytoplasm of the proximal tubules (Figure 5b).

In the 37th developmental week in the dysplastic kidneys (4th developmental period), cystic dilations of the proximal tubules and increased connective tissue are observed. An irregular KLK6 signal is observed only in the apical cytoplasm of some proximal tubular cells, while it is present in the dilated lumen of the proximal tubules in the form of detritus, as the apical cytoplasm seems to be shedding into the lumen. Acetyl-α-tubulin stains the primary cilia, which in the proximal tubules appear irregular and branching at places, forming dense accumulations. The merging of the images reveals the co-expression of KLK6 and acetyl-α-tubulin only in places where KLK6 is expressed in parallel to acetyl-α-tubulin (Figure 5c).

In the 37-week-old dysplastic kidney (4th developmental period), the dilated proximal tubules are observed in the kidney cortex. A less intense KLK6 signal characterizes the apical parts of the dilated proximal tubules as the remaining apical cytoplasm is observed as shed in the tubular lumen. A weak KLK6 signal is also observed in the form of fine grains in the kidney stroma. An AQP1 signal is observed in the basolateral cytoplasm of the dilated proximal tubules. After the merging of the images (KLK6, AQP1, and DAPI), there is no overlapping of the antibodies as each of them is localized in its cellular compartment (KLK6 in the apical part, while AQP1 is in the basal two-thirds) (Figure 5d).

In the same dysplastic kidney tissue, AQP2 coarse grains are observed throughout the cytoplasm of many distal tubules, while the cytoplasm of the proximal dilated tubules is primarily free of AQP2 expression.

The co-localization of the KLK6 and AQP2 stain with DAPI reveals the absence of their co-expression as KLK6 localizes in the apical cytoplasm of the proximal tubules, while AQP2 is located in the distal tubules (Figure 5e).

### 3.4. Expression Dynamics of KLK6 in Normal Fetal Kidney Tissues and Comparison of the Area Percentage of KLK6-Positive Cells Between Control and Kidneys Affected with Congenital Anomalies of the Kidney and Urinary Tract (CAKUT)

**Figure 6:**
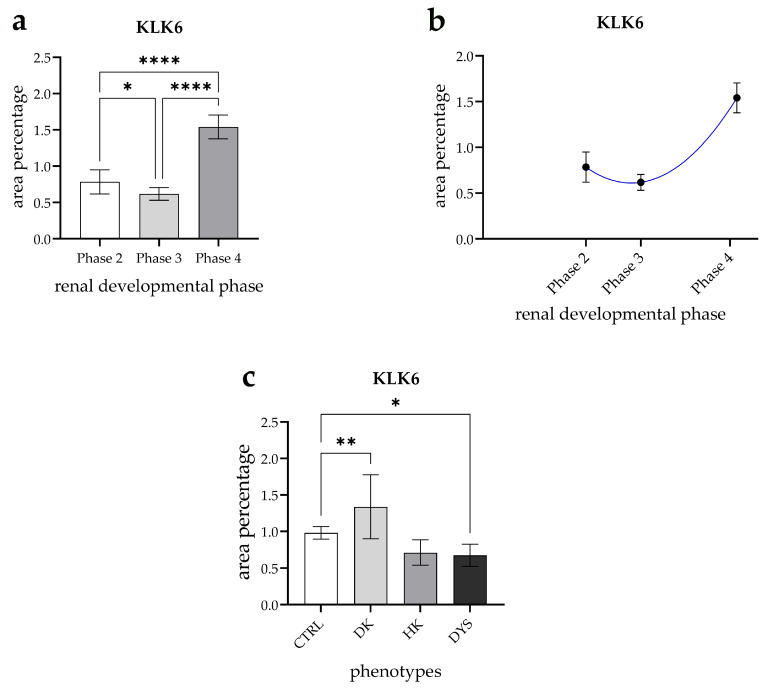
(**a**) The expression of KLK6 in the healthy kidneys throughout the developmental phases 2, 3, and 4. Data are presented as the mean ± SD (vertical line) and analyzed using an ordinary one-way ANOVA test followed by a Tukey’s multiple comparison test. (**b**) The expression dynamics of kallikrein 6 (KALK6) are shown by linear and nonlinear regression modeling of the area percentages through the developmental phases in the cortex of normal fetal kidney tissues during developmental phases 2, 3, and 4. (**c**) The KALK6 area percentages in the fetal tissues of the normal developing kidneys (CTRL), duplex kidneys (DKs), horseshoe kidneys (HKs), and dysplastic kidneys (DYSs). Data are presented as the mean ± SD (vertical line) and analyzed using an ordinary one-way ANOVA test followed by a Tukey’s multiple comparison test. Significant differences are indicated by * *p* < 0.05, ** *p* < 0.01, and **** *p* < 0.0001. At each time point, ten representative pictures were assessed.

KLK6 expression in healthy kidneys exhibited statistically significant variation across the developmental phases (*p* < 0.05, Figure 6a). The expression levels in phase 3 were significantly lower than in phase 1 (*p* < 0.05), while phase 4 demonstrated the highest expression relative to all the other phases (*p* < 0.0001) (Figure 6a).

Furthermore, KLK6 expression followed a quadratic trajectory, reaching its peak in phase 4 (R^2^ = 89.61%, Figure 6b).

A comparison of KLK6 expression across kidney phenotypes revealed significant differences in the area percentage. The KLK6-positive cell area was significantly higher in duplex kidneys (DKs) compared with control kidneys (CTRL) (*p* < 0.001, Figure 5a). In contrast, KLK6 expression was significantly lower in dysplastic kidneys (DYSs) than in controls (*p* < 0.05, Figure 6c).

## 4. Discussion

Congenital anomalies of the kidney and urinary tract (CAKUT) refer to structural and functional defects affecting the kidneys, collecting system, bladder, and urethra [10]. Epidemiological studies indicate that the prevalence of CAKUT in the general population ranges from 0.04% to 1%, with even higher rates observed in preterm infants [12]. The clinical manifestation of CAKUT encompasses a broad spectrum of anomalies, including renal agenesis, horseshoe kidneys, and hypoplastic and dysplastic kidneys, which are included in this study [10]. While some cases remain asymptomatic, others may lead to progressive renal dysfunction, increasing the risk of chronic kidney disease (CKD) over time [56]. As kidney function declines, various pathological processes emerge, including impaired protein filtration (proteinuria), apoptosis, inflammatory cell infiltration, and extracellular matrix accumulation in the interstitium [57]. Additionally, oxidative stress plays a crucial role in renal injury by driving the expression of proinflammatory and profibrotic molecules, further exacerbating disease progression [58].

Among the key regulatory pathways mediating these processes, the kallikrein–kinin system (KKS) is vital as a complex multi-enzymatic network [59]. This system involves tissue and plasma kallikreins, which generate kinins [27,60]. Kinins are traditionally recognized as peptides involved in vascular and inflammatory processes [59]. The renal KKS is involved in both intrarenal and extrarenal complex events, such as regulating the extracellular volume, maintaining blood pressure, and controlling sodium and water excretion, along with renal vascular resistance and renin release [27,59]. Abnormalities in the KKS have been linked to major diseases, including hypertension [61] and diabetes mellitus [62].

Although the classical kallikrein–kinin system (KKS), primarily driven by KLK1, is well established in many renal regulatory mechanisms, the specific contribution of KLK6 to these processes remains unclear. KLK6 is not traditionally considered part of the classical KKS but is recognized for its role in tumorigenesis and inflammatory pathways [28]. Recent research has identified KLK6 as a promising prognostic biomarker in various cancers, while the co-expression of KLK6 and KLK10 has been suggested as a potential indicator of survival outcomes in pancreatic ductal adenocarcinoma [63]. Additionally, KLK6 overexpression in ovarian [64,65,66], breast [67], colorectal [68], and bladder urothelial cancer tissues [69] has been associated with a poor prognosis.

In renal cell carcinoma (RCC), KLK6 expression varies by subtype and tends to be elevated in more aggressive tumors, correlating with advanced staging and poorer survival, making it a potential biomarker for RCC subtyping [24,25]. Beyond cancer, KLK6 has been explored as a biomarker in renal impairment. Clinical studies suggest that circulating KLK6 levels positively correlate with serum creatinine concentrations in patients with renal failure, indicating its potential utility in assessing kidney function [70]. However, the precise mechanistic role of KLK6 in kidney physiology and disease progression remains speculative, necessitating further investigation.

The results of this study reveal that the expression pattern of KLK6 undergoes distinct spatiotemporal changes throughout normal human kidney development. While KLK6 expression is consistently observed in the proximal convoluted tubules across all the examined developmental phases, its subcellular localization varies over time. Initially, KLK6 is predominantly localized to the basal and lateral cytoplasm of proximal tubule cells. However, as development progresses, its localization shifts toward the apical cytoplasm. By the fourth developmental stage, KLK6 expression becomes further restricted to the apical domain, with a concomitant reduction in its presence within the basal and lateral cytoplasm. This expression pattern partially aligns with the findings of Gabril et al., who reported strong cytoplasmic KLK6 expression in both the proximal and distal tubules, weak expression in the mesangium, and no detectable expression in Bowman’s capsule in the normal human kidney. We hypothesize that KLK6 localization to either the luminal or basal plasma membrane may strategically position it to act on its substrates in either the tubular fluid or the peritubular space, as already noted in a study by Vio et al. when exploring KLK1 in the distal tubule [71]. Considering our results, the main targets of KLK6 activity during early kidney development (KLK6 localized basally) might be in the peritubular space, possibly the extracellular matrix (ECM) components, while the main substrates in mature kidneys (KLK6 localized apically) could be in the tubule lumen.

A quantitative analysis revealed that KLK6 expression in healthy kidneys varied significantly across the developmental phases and followed a quadratic trajectory. Expression levels in phase 3 were considerably lower than in phase 1, whereas phase 4 exhibited the highest KLK6 expression relative to all the other phases. This marked increase in the final developmental phase aligns with its strong expression in mature kidneys, suggesting a potential role in proximal tubule differentiation and functional maturation. As a serine protease, KLK6 facilitates the degradation of ECM components, a process that is crucial in tissue remodeling during nephron and tubular formation, as demonstrated in studies of its role in other organs [14]. The increased expression of KLK6 in the later developmental phases underscores its significance in refining the structural framework necessary for mature kidney function. Additionally, KLK6 activates proteinase-activated receptors (PARs), such as PAR1 and PAR2, which are integral to cellular processes, including differentiation and proliferation. This activation modulates the signaling pathways that influence tubular differentiation and maturation, thereby contributing to the development of functional renal tubules [14].

Ciliogenesis in developing and postnatal human kidneys appears to play a key role in regulating cell proliferation, differentiation, apico-basal polarity, and tubular lumen formation. Staining with acetyl-α-tubulin, a marker for primary cilia, revealed elongated cilia on the apical surfaces of all tubular cells and glomeruli, with a particularly high abundance in distal tubules, consistent with our previous research [34,35,72]. Additionally, immunoexpression patterns show a gradual decline in acetylated α-tubulin levels during normal kidney development, aligning with prior findings [34,35]. Notably, KLK6 was co-expressed with acetyl-α-tubulin on the apical surfaces of the proximal tubular cells. KLK6 was also co-expressed with AQP1 in the proximal convoluted tubule cells, though localized in distinct cellular compartments. In contrast, no co-expression was observed between KLK6 and AQP2, as AQP2 was exclusively localized to some distal convoluted tubules and connecting tubules in the renal cortex.

KLK6 expression varied significantly across different kidney phenotypes, with distinct differences in the proportion of the KLK6-positive cell area. Duplex kidneys (DKs) exhibited a considerably higher KLK6-positive area compared with control kidneys (CTRLs), suggesting an association between increased KLK6 expression and the structural anomalies that are characteristic of duplex kidneys. Structural abnormalities, such as ureteral obstruction, hydronephrosis, or altered nephron distribution, could create localized hypoxia, inflammation, or mechanical stress in duplex kidneys, which may upregulate KLK6 as part of a reparative or inflammatory response.

Compared with normal kidney tissue during the second phase of kidney development, KLK6 expression in dysplastic kidneys (DYSs) is predominantly localized to the apical cytoplasm of the proximal tubules. Notably, DYSs exhibited significantly lower KLK6 expression than the controls. This reduction corresponds with the disrupted renal architecture that is characteristic of dysplastic kidneys, where distorted cilia—varying in number, size, and shape—were observed within proximal tubule microcysts. Additionally, proximal tubule segments displayed abnormally elongated cilia, resembling the morphological features previously described in multicystic dysplastic kidney (MCDK) cases by Saraga et al. [72].

Our previous studies revealed that dysplastic kidneys demonstrated initially elevated expression and a subsequent decline [35]. This pattern suggests an initial compensatory response to structural and developmental challenges, followed by a failure to sustain cytoskeletal stability [35]. Proteolytic enzymes, such as KLK6, play a key role in remodeling the ECM, a process that is essential in maintaining the structure and function of cilia. The downregulation of KLK6 may exacerbate the decline in ciliary integrity by impairing proteolytic activity and disrupting epithelial remodeling, both of which are vital for normal kidney development. Without adequate KLK6 activity, the proximal tubule cells may fail to maintain cytoskeletal integrity, leading to the eventual loss of acetylated α-tubulin-positive cells. This decline could further compromise the structural stability of the nephrons and exacerbate the dysplastic phenotype.

In contrast to normal kidneys, in CAKUT kidneys, AQP1 demonstrated an irregular localization and a weaker expression in the proximal tubules, especially dilated ones, while AQP2 expression, normally found only apically and in a subset of distal tubules, was present in some dilated proximal tubules and throughout the cytoplasm of many distal tubules. Our results correspond with formerly published observations regarding AQP1 and AQP2 and their role in cyst formation and development. Wang et. al. described how the overexpression of AQP1 inhibited cyst growth in a MDCK cyst model, while the deletion of *AQP1* promoted cyst development in an embryonic kidney and a PKD mouse model [73]. On the other hand, the overexpression of AQP2 has been described to enhance cyst enlargement [74]. Additionally, Gao et al. have demonstrated in mouse models that AQP2-positive progenitor cells have a crucial role in the maintenance and continuous regeneration of distal tubule cells in response to injury [43], which would explain the increase in AQP2-positive distal tubule cells in our CAKUT samples.

Our previous studies in the same tissue cohort investigated the spatio-temporal expression patterns of several candidate genes implicated in CAKUT pathogenesis during human fetal kidney development. Specifically, we analyzed FGFR1, FGFR2, and RIP5 expression across developmental stages (22nd to 41st week) in both healthy and malformed kidneys (duplex, hypoplastic, and dysplastic) using immunofluorescence and RT-qPCR [47]. These analyses revealed marked differences between the CAKUT and control tissues. FGFR1 displayed altered nuclear localization and dysregulated expression dynamics in dysplastic kidneys, while FGFR2 expression was significantly upregulated in CAKUT samples, possibly indicating a compensatory ERK1/2-mediated repair response. RIP5 expression followed a developmental pattern in controls but was diminished in CAKUT kidneys, suggesting impaired maturation and disrupted homeostatic signaling.

In a separate line of investigation, we also assessed the expression of EDA2R, PCDH9, and TRAF7 in both the cortical and medullary regions of control and CAKUT-affected kidneys [46]. EDA2R was elevated in dysplastic samples, potentially reflecting increased apoptotic activity. Conversely, PCDH9 expression was reduced in horseshoe kidneys, possibly due to its role in regulating cell migration. TRAF7 expression was consistently decreased across various CAKUT phenotypes, pointing to a broader disruption in endothelial development and ciliogenesis. Collectively, these findings emphasize the importance of these candidate genes in both normal nephrogenesis and the molecular mechanisms underlying CAKUT.

The main limitation of this study lies in its observational nature. Given that our samples consist of archived paraffin-embedded and formalin-fixed human fetal material, we could not conduct quantitative protein expression analyses using flow cytometry or Western blotting methodologies. IF was used as a surrogate approach to assessing protein expression. Furthermore, the study is constrained by a relatively small sample size derived from a single hospital center, which may limit the generalizability of our conclusions. Another point is that our investigation focused exclusively on protein expression without incorporating a complementary mRNA expression analysis, which could have provided further insights into regulating the studied proteins.

Despite these limitations, our study presents significant value due to the rarity of the human material analyzed. To our knowledge, this is the first study to characterize the immunoexpression patterns of KLK6 in normal human kidney development and CAKUT. Further research is required to ascertain whether KLK6 directly influences renal function through protease-mediated signaling or extracellular matrix remodeling, or if its altered expression merely reflects secondary pathological changes.

## 5. Conclusions

The findings of this study highlight the dynamic expression pattern of KLK6 throughout kidney development, with distinct spatial and temporal variations in its localization. The observed shifts in KLK6 distribution from the basal and lateral cytoplasm toward the apical domain of proximal tubular cells suggest its potential role in tubular differentiation and functional maturation. Furthermore, the increased KLK6 expression in the later developmental phases aligns with its involvement in extracellular matrix remodeling, a process that is essential in nephron formation and structural integrity.

The differential KLK6 expression observed in CAKUT phenotypes, particularly in duplex and dysplastic kidneys, emphasizes its potential role in renal pathophysiology. Elevated KLK6 expression in duplex kidneys may reflect a compensatory mechanism in response to structural abnormalities. Conversely, the reduced KLK6 expression in dysplastic kidneys, coupled with ciliary defects and a disrupted renal architecture, suggests a failure to sustain cytoskeletal stability and epithelial remodeling, ultimately exacerbating disease progression.

Notably, the distinct spatial and temporal expression patterns of KLK6 observed in our study raise the possibility that it could serve as a biomarker for the early detection of developmental kidney anomalies, including CAKUT. Its detection in tissue may eventually be complemented by non-invasive approaches, such as the analysis of KLK6 levels in serum or urine, which could offer novel avenues for early diagnosis, disease monitoring, or the stratification of congenital renal pathologies.

In addition to its potential diagnostic utility, KLK6 may hold therapeutic relevance. Should future studies confirm its direct involvement in pathological remodeling or inflammatory signaling, KLK6 could emerge as a candidate for targeted modulation. Inhibitors of related kallikrein family members are already under investigation in other disease contexts [75,76,77], and similar strategies might be explored for KLK6 in nephrology. Understanding its specific substrates, downstream signaling partners, and regulation during nephrogenesis will be crucial for evaluating its feasibility as a therapeutic target [78,79]. Ultimately, these insights could pave the way toward novel diagnostic and therapeutic strategies in congenital and acquired renal diseases.

## Figures and Tables

**Table 1:** The human fetal kidney samples (*n* = 37) analyzed in the study.

Phenotype	Developmental Phase	Renal and Related Pathology	Gestational Phase	Number of Samples
Normal kidneys (CTRL)	Ph2	N/A	15 dw	1
		N/A	16 dw	2
		N/A	17 dw	2
		N/A	18 dw	2
	Ph3	N/A	23 dw	1
		N/A	24 dw	1
		N/A	28 dw	2
		N/A	29–30 dw	1
	Ph4	N/A	32 dw	1
		N/A	35 dw	1
		N/A	37 dw	1
		N/A	38 dw	2
		N/A	1 m	1
		N/A	1.5 y	1
		N/A	7 y	1
Horseshoe kidney (HK)	*Ren concreatus arcuatus*, *Cystae multiplices corticales*		22 dw	1
	*Ren concreatus arcuatus*, *Tetras Fallot*		26 dw	1
	*Syndroma Edwards*, *Ren arcuatus*		30–31 dw	1
	*Syndroma Edwards*, *Ren arcuatus*		34 dw	1
Dysplastic kidney (DYS)	*Megaureter lateris dextri*, *Dysplasia renis*		21 dw	2
	*Renis dysplastica cysticus lateralis sinistri*, *agenesia renis dextri*			
	*Dysplasia multicystica renis dextri*, *Cystes parvae focales*		27 dw	1
	*Cystes multiplices renum praecipue*, *Dylatatio cystica calycium*, *Teratoma sacrococcygeale*		28 dw	2
	*Renes multicystici dysplastici*, *Megaureter bilateralis*, *Stenosis ostia ureteris bilateralis*, *Syndroma Potter*, *Syndroma Down*		33–34 dw	2
	*Renes dysplastici cystici*, *Syndroma Potter*		35 dw	1
	*Dysplasia renis multicystica bilateralis*			
	*Agenesis renis dextri et dysplasia renis sinistri cum ureter duplex*		37 dw	1
	*Dysplasia hypoplastica*, *renis bilateralis*, *Syndroma Down*, *Syndroma Potter*		38 dw	1
	*Dysplasia renis*, *Syndroma Potter*		39 dw	1
Duplex kidneys (DK)	*Ureter duplex lateris dextri*		24 dw	1
	*Ureter duplex lateris sinistri*		30 dw	1
	*Pyelon et ureter duplex bilateralis*		41 dw	1

dw—developmental week; m—month; y—year; N/A—not applicable.

**Table 2:** Primary and secondary antibodies used for immunofluorescence staining.

Antibody		Catalog Number	Host	Dilution	Source
Primary	Anti-kallikrein 6 antibody	ab191281	Goat	1:100	Abcam (Cambridge, UK)
	Anti-acetyl-α-tubulin (Lys40) (6-11B-1)	12152S	Mouse	1:500	Cell Signaling Technology (CST), (Danvers, MA, USA)
	Aquaporin 1/AQP1 Antibody (B-11)	sc-25287	Mouse	1:50	Santa Cruz Biotechnology (Dallas, TX, USA)
	Aquaporin 2/AQP2 Antibody (E-2)	sc-515770	Mouse	1:50	Santa Cruz Biotechnology (Dallas, TX, USA)
Secondary	Alexa Fluor^®^ 488 AffiniPure™ Donkey Anti-Goat IgG (H + L)	705-545-003	Donkey	1:400	Jackson Immuno Research Laboratories, Inc., (Baltimore, PA, USA)
	Rhodamine Red™-X (RRX) AffiniPure™ Donkey Anti-Mouse IgG (H + L)	715-295-151	Donkey	1:400	Jackson Immuno Research Laboratories, Inc., (Baltimore, PA, USA)

## Data Availability

The original contributions presented in this study are included in the article. Further inquiries can be directed to the corresponding author.
